# Separation of the field source and characterization of the deep and shallow tectonics of the northwestern margin of the Sichuan-Yunnan rhombic massif

**DOI:** 10.1038/s41598-025-29828-z

**Published:** 2025-11-28

**Authors:** Long Qinhong, Wu Guiju, Xi Yufei, Zhang Rui

**Affiliations:** 1 Key Laboratory of Earthquake Geodesy, Institute of Seismology, CEA, Wuhan, China; 2 Institute of Hydrogeology and Environmental Geology, Chinese Academy of Geological Sciences, Shijiazhuang, China

**Keywords:** Bouguer gravity anomaly, 2-D discrete wavelet transform, Radial logarithmic power spectrum, Sichuan-Yunan block, Geodynamics, Geophysics

## Abstract

The Sichuan-Yunnan block, characterized by complex geological formations and persistent high-intensity seismicity, remains a focal domain for contemporary geodynamic research. To investigate the fracture depth of the northern Sichuan-Yunnan block and the southeastern part of the Qiangtang block, CRUST1.0 was employed to fit the trend items in the study area and to refine the gravity field model with measured data as control points. A two dimensional discrete wavelet transform analysis method is applied to separate the field sources from the obtained high-precision grid data. Additionally, radial logarithmic power spectrum analysis is utilized to estimate the average equivalent source depth of the abnormal geological bodies corresponding to each order wavelet, thereby extracting both transverse and longitudinal tectonic characteristics of the crust in the region. The results reveal that the Bouguer gravity anomaly within the study area exhibits a general pattern of low values in the northern region and high values in the southern areas. Major faults, including the Bianba-Luolong fault, Nujiang fault, Lancangjiang fault, Batang fault, Jinshajiang fault, Yushu-Ganzi fault and Ganzi-Litang fault are all deep and large faults, with respective depths of 38.9 km, 52.9 km, 52.9 km, 52.9 km, 38.9 km, 52.9 km, 52.9 km. This study provides physical insights into the crustal structure distribution of the northern Sichuan-Yunnan rhombohedral block.

## Introduction

The junction area between the Sichuan-Yunnan block(SYB) and Qiangtang block.

(QTB) is located at the southeast of Tibetan Plateau(TP), in an area of intense deformation. The eastward flow of material from the TP provides tectonic stress, and the gravity anomalies of the crust are very complex, making it one of the regions with the most significant tectonic activity in mainland China^1^. As a result of the active plate tectonic movement in the region, there are many fractures in the study area, with complex stress accumulation and frequent earthquakes. There are historical records that in April 1989, a strong earthquake swarm of Ms6.7 occurred in Batang, Sichuan Province, and before that, several earthquakes of Ms6 or higher had occurred in Batang. Stresses released at the fracture surfaces cause earthquakes. The study of the depth of fracture extension is important for understanding the causes of earthquakes, their mechanisms, and their destructiveness^2,3^.

Since 1975, when geophysical researchers made comprehensive geophysical observations of the TP , many scholars have conducted a lot of research on the study area^4^. The QTB is in the upper central part of the TP and the southern part of the Songpan-Ganzi block. It is mainly composed of Paleozoic and Mesozoic metamorphic and sedimentary rocks. The QTB was drifted from Gondwana in the Late Paleozoic and collided with the Songpan-Ganzi block in the Early Jurassic of the Late Triassic^5^. Song et al. established the Paleozoic–Mesozoic stratigraphic framework of the Tethys orogenic system in Qinghai-Tibet by analyzing the depositional background of the oceanic plate and the stratigraphic sequence. Thus, the evolution of the paleogeographic pattern was revealed^6^. Rigid block models suggest that continental deformation occurs primarily on strike-slip faults, which are the boundaries between tectonic blocks^7–9^.

Li et al. analyzed the crustal deformation characteristics of the southeast of TP using GPS data. Their results show that the Xianshuihe-Anninghe fault has strong localized shear strain, consistent with tectonic extrusion along the rupture, which ends in southern Yunnan and then shifts to crustal extension and contraction^10^. Xu et al.studied the kinematics of the Jinshajiang fault zone and its slip rate. It provides a basis for the dynamic mechanism of tectonic deformation on the southeastern margin of the TP^11^. Qiao et al. conducted an analysis of the GPS results from 1998 to 2002, which revealed that the boundary rupture in the SYB exerts a substantial absorbing effect on plate motion and rotation. This, in turn, imposes limitations on the further diffusion of materials on the eastern side of the TP^12^. The results of the studies indicate the presence of a significant gravity anomaly of low value in this region^13^.Gao et al. found that the strata exposed in the northern part of the SYB are almost all Triassic, and the density of the overall orogenic zone is relatively low, with the difference between the upper and lower densities reaching 0.31 ~ 0.8 g/cm^3^. Furthermore, they identified the presence of a low-velocity layer within the crustal depths of 15–30 km, with a thickness ranging from 8–10 km. This layer exhibited a P-wave velocity of 5.8 km/s, indicative of its low velocity characteristics^14^.

The Moho depth exhibited a general trend of shallowness in the southeast and depth in the northwest within the study area. While the general trend was comparable, the specific Moho surface depths in disparate areas demonstrated variability^15^. The crust of the TP is divided into three distinct layers: upper, middle, and lower. The middle layer is characterized as a low-resistance zone. A high conductivity zone or laminar flow has been identified in the mid-crust (20–40 km) of the study area, which extends horizontally from the TP to the southwest of China. The main areas of laminar flow are located in the inter-fracture zones^5,16–18^. Zhou et al. posited that the upwelling of hot mantle material due to subsidence resulted in the gradual replacement of the lithospheric mantle material of the SYB by new mantle material from south to north^19^.

The depth of the Moho surface undulation and the extent of the deep and large fractures in the study area are subjects of considerable controversy. In this paper, we seek to ascertain the extent of each primary fracture within the study area. To this end, we employ 2-D discrete wavelet transform(DWT2D) and radial logarithmic power spectral analysis to determine the initial rupture depth and the rupture termination depth of each fracture. By leveraging these methodologies, we aim to substantiate the Moho surface depth through the fracture rupture depth.

## Regional tectonic setting

**Fig. 1:**
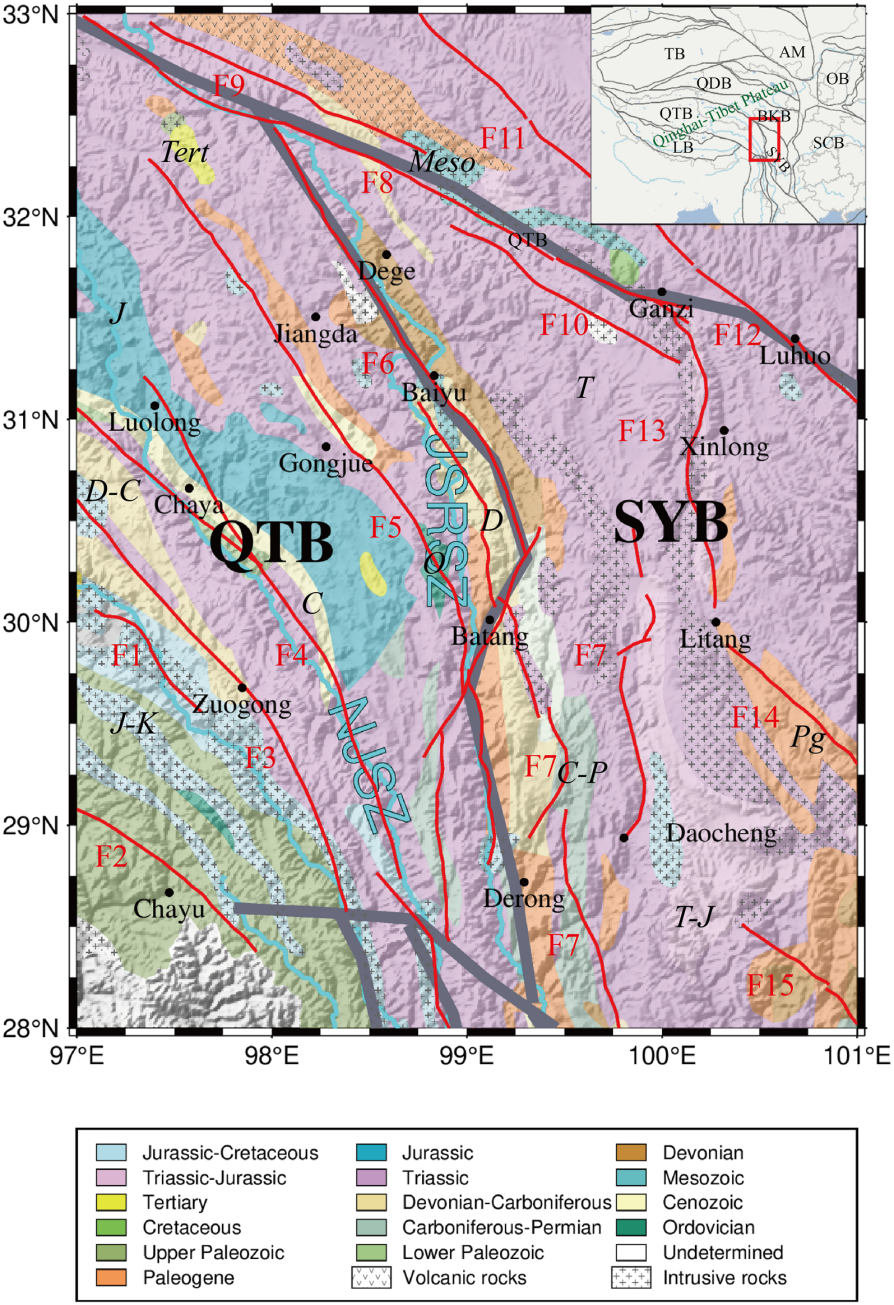
Geotectonic map of the study area. Plate boundary are in gray, LB: Lhasa Block; QTB: Qiangtang block, QDB: Qaidam Block; TB: Tarim Block; AM: Alxa Massif; BKB: Bayan Kala Block; SYB: Sichuan-Yunnan block. OB: Ordos Basin; SCB: South China Block; Black solid dots are county names. Red lines are faults, and the following are fracture pronouns. F1: Bianba-Lolong fault, F2: Jiali fault, F3: Nujiang fault, F4: Lancangjiang fault, F5: Zigasi-Deqin fault, F6: Dedeng-Batang-Riyu fault, F7: Jinshajiang fault, F8: Yushu-Ganzi fault, F9: Haxiu-Chaxika fault, F10: Dalangsonggou fault, F11: Wudaoliang-Changshagongma fault, F12: Xianshuihe fault, F13: Ganzi—Litang fault, F14: Litang fault, F15: Boko fault. NJSZ: Nujiang Suture Zone JSRSZ: Jinsha River Suture Zone.

The study area of this paper has a complex geological structure (Fig. 1), which brings together the SYB, QTB and the southeastern part of Songpan-Ganzi block. Most of the area is above 3500 m in altitude^20^. The geological map data for this article are provided by the United States Geological Survey(USGS) at a resolution of 1:5000000^21^. The study area is situated at the junction of tectonic plates, where numerous faults are present. As a result of fault activities, earthquakes have occurred frequently in this region over the past decade. This paper compiles an earthquake catalog (Table 3) for all earthquakes with magnitudes of 6 or greater in the study area since records began, with data sourced from Earthquake Cataloging in China. The southeastern region of the TP is predominantly characterized by Paleozoic and Mesozoic formations, with the Triassic being the most prominent, followed by a minor presence of Jurassic and Lower Paleozoic strata, etc. The predominant stratigraphic lithology of the Triassic is intrusive rock formations (Fig. 1). A minor presence of volcanics is observed in the northeast distribution of the Haxiu-Chakaxi fault(F9). Fractures in the region from west to east are the Bianba-Lolong fault(F1), the Jiali fault(F2), the Nujiang fault(F3), the Lancangjiang fault(F4), the Zigasi-Deqin fault(F5), the Dedeng-Batang-Riyu fault(F6), the Jinshajiang fault(F7), the Yushu-Ganzi fault(F8), the Haxiu-Chakaxi fault(F9), and the Dalangsonggou fault(F10),Wudaoliang-Changshagongma fault(F11), Freshwater River fault(F12), Ganzi-Litang fault(F13), Litang fault(F14), and Boko fault(F15).This paper will focus on seven of these fractures: F1, F3, F4, F6, F7, F8, and F13. Fractures are predominantly north-west oriented, as illustrated by the F3, the F4, and the F8. However, the F6 and the F7 exhibit different characteristics, as detailed in Table 1.

**Table 1:** Fracture strike and sliding rate in the study area.

Fault name	Strike	Slip rate
Bianba-Lolong fault(F1)	NW	1–1.7 mm/a
Jiali fault(F2)	N60°W	~ 4 mm/a
Nujiang fault(F3)	NWW-NW	~ 3.2 mm/a
Northern section of Lancangjiang fault(F4)	NW	3.5 + 1.1/-0.8 mm/a
Zigasi-Deqin fault(F5)	NW	-
Dedeng-Batang-Riyu fault(F6)	N30°E	2 ~ 4 mm/a
Jinshajiang fault(F7)	SN	5 ~ 7 mm/a
Yushu-Ganzi fault(F8)	NW	5 ~ 7 mm/a
Haxiu-Chaxika fault(F9)	NW	-
Dalangsonggou fault(F10)	NW	-
Wudaoliang-Changshagongma fault(F11)	N45°W	2.4 ± 0.2 mm/a
Xianshuihe fault (Luhuo section)(F12)	NW	9.13 mm/a
Ganzi—Litang fault(F13)	NNW	-
Litang fault(F14)	N40°-50°W	4.0 ± 1.0 mm/a
Boko fault(F15)	-	-

## Bouguer gravity anomaly in the study region

The EGM2008 model represents a significant milestone in the field of global gravity field modeling. Notably, it is the first ultra-high-order model, integrating GRACE satellite data and global 5-arc-mean free-air gravity anomaly data. Additionally, it provides comprehensive error estimates and uncertainty analyses, resulting in a highly accurate model across all wavelength ranges^22,23^.In this paper, the measured regional Bouguer gravity anomaly(BGA) data are used as the control point, the CRUST1.0 model data are used to fit the trend term in the study area, and the terrain-corrected EGM2008 BGA data are fused into a high-precision gridded data set with a resolution of 2.5 km × 2.5 km. This method allows for the determination of the extension depth of the deep rupture. The DWT2D and the radial logarithmic power spectrum analysis are employed to achieve this objective.

**Fig. 2:**
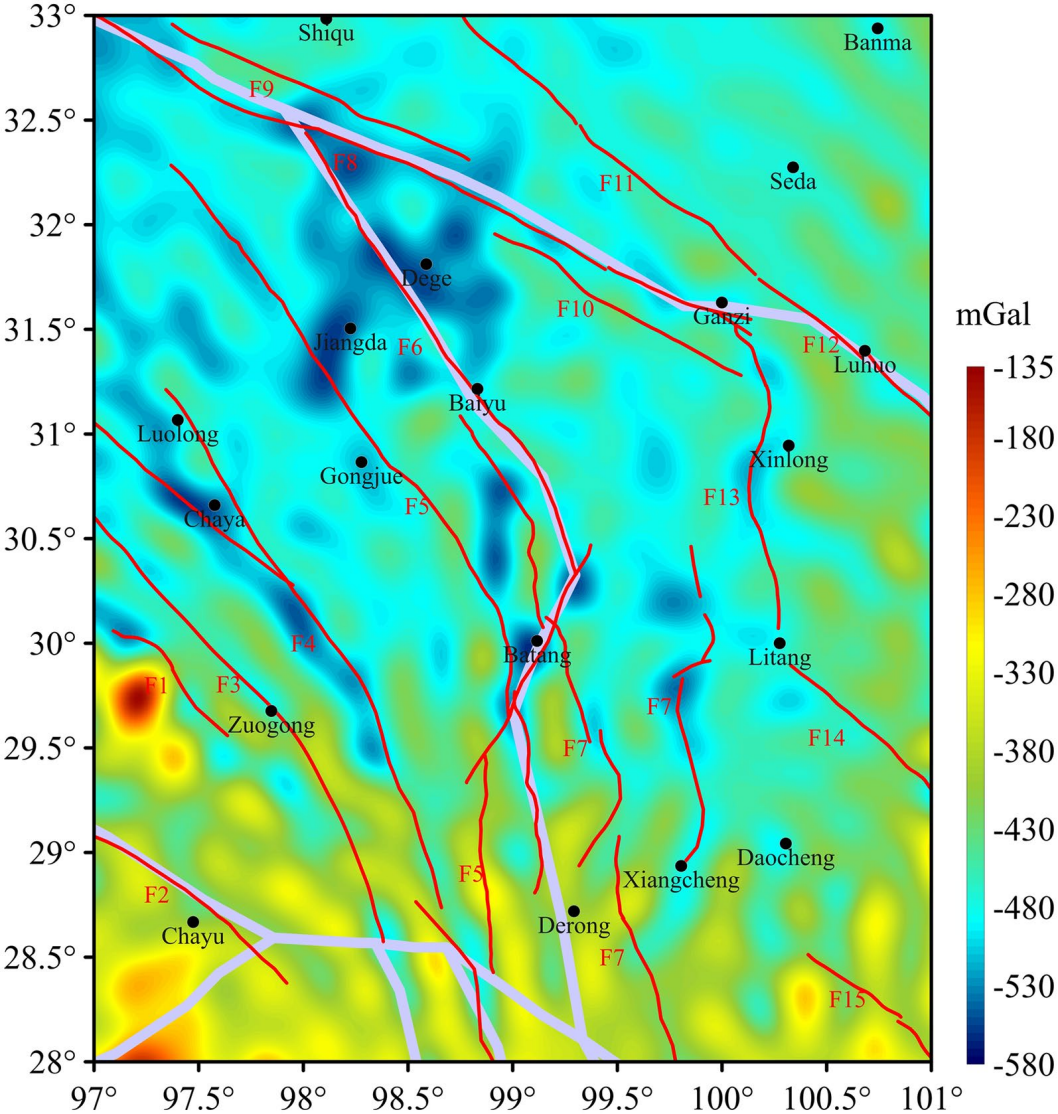
Bouguer Gravity anomaly in the study area. Magenta dots indicate the location of earthquakes. Small dots represent earthquakes of magnitude 6 or above but below magnitude 7, while large dots represent earthquakes of magnitude 7 or above.

In the study area, the low negative value of the BGA is predominant (Fig. 2), with an amplitude ranging from -580 to -135 mGal. The distribution of the BGA is characterized by a general trend of lower values in the northern region and higher values in the southern region. The distribution of the F1, F2, and F3 fractures in the southwestern part of the study area aligns predominantly along the BGA low value. Notably, F1 corresponds to the western boundary of the Nujiang Suture Zone(NJSZ), a Holocene active fracture. The F2 coincides with the southern boundary of the QTB, which is the southern boundary of the TP where subsurface material escapes outward. The east and west sides of this rupture are dominated by BGA high-negative anomalies, and Chaya, Jiangda, Dege, and Batang are the obvious low-negative anomaly areas in the study area, which are lower than -530 mGal. The several ruptures, F4, F5, and F6, are mainly spread out along the low-value of the BGA. The F4 is distributed along the BGA low-value zone, and the 2013 Changdu Ms6.1 earthquake occurred in the upper right of Zuogong in the middle section of F4. The F5 represents the boundary fracture of the QTB and the Jinsha River Suture Zone (JSRSZ). North of Gongjue, the fault is primarily distributed along the BGA low value, while south of Gongjue, the fault is distributed along the BGA transition zone. The F6, a dextral strike-slip active fracture, traverses the Jinsha River tectonic belt in an oblique manner. This fracture is a consequence of internal land deformation and is delineated by Baiyu. In the northern sector, the distribution of the F6 fault aligns with the low-value BGA, while in the southern sector, it is predominantly concentrated along the BGA low value. The activity of this fault has been evident since the Late Quaternary period, with a notable occurrence recorded during the 1870 Batang Ms7.0 earthquake, There was an 1870 Batang Ms7 earthquake, located at F6^24,25^. The F8, F9, and F10 are three parallel faults that are distributed along the eastern boundary of the SYB. The F13, situated to the left of Xinlong, exhibits a linear distribution along the BGA transition zone in the region, manifesting as an elongated anti-S configuration.

The southeastern region of the TP has experienced significant crustal fragmentation due to the substantial extrusion of the Indian plate, resulting in the development of numerous deep faults. Studies have demonstrated that a portion of the molten material within the southeastern region of the TP has disseminated eastward through crustal channel flows, thereby impeding the plateau’s vertical rise^26–29^. The SYB is a natural “experimental base” for studying the laws of seismic activity and geotectonic relationships due to its frequent and intense crustal activities. The findings of the DWT2D reveal substantial variations in the crustal structure between the northern and southern regions of the SYB, which can be categorized into two sub-blocks based on the presence of faults^30^. Along the JSRSZ, the rock structure near the Batang fault is very fragmented, and mudslides, landslides, and other natural geologic hazards have occurred within three kilometers on either side of the fault in about half of the cases. Since the inception of recorded history, the Batang fault has been impacted by seven significant earthquakes of Ms6 or higher, the most notable of which was the Batang earthquake of 1870, which resulted in the loss of over a thousand lives. The NJSZ can be divided into the northern section, the middle section and the southern section from northwest to southeast. The middle section is the main one in the study area. Within the confines of the study area, the Bangda fault exhibits significant interruptions, with frequent occurrences of strong earthquakes in the central segment. These seismic events extend beyond historical tremors; a Ms5.5 earthquake also occurred in 1950, underscoring the area’s seismic activity^31^. As depicted in Fig. 2, earthquakes with a magnitude of 6 or higher in the region all occur near faults or in the BGA transition zone, and the distribution follows a certain pattern. This will be discussed later in this article.

## Field-source separation theory

### 2-D discrete wavelet transform

Following over a century of development, gravity methods have established a comparatively robust theoretical and technical system for investigating the composition and alterations of the Earth’s crust. The gravity anomalies are defined as the superimposed effect of gravity anomalies of geologic bodies of different depths, sizes, and densities in the subsurface. In order to study the gravity anomaly of a specific geologic body, it is first necessary to separate the gravity anomaly caused by the geologic target body from the superimposed anomalies. This process enables the inversion and interpretation of the gravity anomaly. A comparison of gravity field observation with other geophysical methods reveals several advantages. Firstly, the equipment and human operation costs are lower. Secondly, the data processing and analysis are relatively simple. Thirdly, the observation time is shorter. Presently, 1:200,000 regional gravity survey studies have been completed in most China’s regions. Consequently, the utilization of gravity field to examine the three-dimensional configuration of the subsurface offers a distinctive advantage.

Presently, DWT2D is among the most frequently utilized methods in gravity anomaly separation. It is a pivotal geophysical data analysis technique. Multi-scale decomposition facilitates the study of gravity anomaly changes in the horizontal direction and the explanation of the distribution of anomalies from different depths, vertically. By selecting an appropriate scale to align with the rupture in the region, the initiation point of the rupture’s rupture can be ascertained^32,33^.

The regional gravity field can be represented by a two-dimensional single-valued real function, assuming a gravity anomaly of

1 $$\Delta g(x,y)=f(x,y)$$

According to the principles of DWT2D and Mallat algorithm. The gravity anomaly decomposition expression can be simplified as

2 $$\Delta g(x,y)={A}_{N}f(x,y)+{D}_{1}f(x,y)+{D}_{2}f(x,y)+\dots +{D}_{N}f(x,y)$$

In Eq. (2)${D}_{1},\dots ,{D}_{N}$ represent the wavelet details generated by the DWT2D, while ${A}_{N}$ denotes the nth order wavelet approximation. A salient property of DWT2D is the low-order detail invariance criterion, which stipulates that the ${D}_{1},\dots ,{D}_{N}$ wavelet details generated by the decomposition remain constant as n increases.

### Bouguer gravity anomaly source depth analysis

A close relationship exists between regional BGA and the burial depth of subsurface anomalies. As the scale of the DWT2D of the BGA increases, the burial depth of the field sources concomitantly increases^34,35^. Two primary methods are typically employed to estimate the burial depth of a field source: the integrated scale inversion method proposed by Yang et al. and the radial logarithmic power spectrum method. In this paper, the radial log power spectrum method is employed to estimate the average equivalent source depth of the anomalous geologic body.

Many scholars have already demonstrated the accuracy of the logarithmic power spectrum to estimate the depth corresponding to the BGA decomposition results, so the depth results obtained in this paper are reliable. Xu et al. experimented with the model using a low-pass wavelet filter and found that wavelet analysis can effectively separate the anomalies. The BGA power spectrum was then leveraged to derive the corresponding subsurface area range, facilitating further investigation into the pre-Cenozoic tectonic^36^. Kadirov et al. examined the power spectrum of gravity anomalies in the Absheron and Shamaki-Gobustan regions. Their findings indicated that the average depth of the field source ranged from 3.2 km to 24.5 km^37^.Erbek and Dolmaz conducted a study that utilized power spectral analysis to examine free-air gravity anomalies and magnetic anomalies in the eastern Mediterranean Sea. Their findings indicated a correlation between gravity anomalies and crustal thickness in this region. Additionally, the researchers employed power spectral analysis of magnetic data, which revealed a correlation between elliptical elongated structures at the Ehlatotseni Seamounts and magmatic basement^38^. Ma et al. employed an adaptive weight function to discern anomalies at varying buried depths. According to the logarithmic power spectrum, the vertical resolution of the inversion results was found to be comparatively enhanced^39^. Javier Sanchez-Rojas’s analysis of the power spectrum of new gravity data from Venezuela indicates average Moho depths of 42, 35, and 40 km, respectively, in the Venezuelan massif, plain, and mountainous regions^40^. Casulla et al. conducted a study to calculate the depths to the Moho surface and bedrock tops in the Sulu Sea. They employed two-dimensional radial log-averaged power spectrum analysis to achieve this objective^41^. Mousa et al. conducted a two-dimensional power spectral analysis of gravity and magnetic data concerning the thickness of the cover of the Western Desert of Iraq and the base structure of the same region. Their analysis yielded results indicating a thickness of 4.5–11.5 km for the cover^42^. Sun et al. discovered a pattern of change in the deformation zone, traversing from the upper crust to the lower crust. The deformation zone exhibited a transition in characteristics from dense and thin in the upper crust to thick and coarse in the lower crust. This observation suggests that the vertical distribution of the deformation zone may be likened to that of a tree, with a thick and coarse trunk in the lower part and dense and thin branches in the upper part^43^.

Assuming that the corresponding burial depth of* D*_*N*_(*N* = *1,2,…,N*) , the result of the DWT2D detail separation of the gravity anomaly, is *h*_*N*,_ the

3 $${D}_{N}=\sum_{m=0}^{M} \sum_{n=0}^{N} {A}_{\omega }^{N}{e}^{i2\pi {\omega }_{N}\left({x}_{m}+{y}_{n}\right)}{e}^{2\pi {\omega }_{N}{h}_{N}}$$

In Eq. (3), ${A}_{\omega }^{N}$ is the amplitude, ${\omega }_{N}$ is the frequency, ${(x}_{m},{y}_{n})$ are the coordinates of the grid point of gravity anomaly ${D}_{N}$, and *m* and* n* are the number of columns and rows of the grid point. Then the amplitude ${A}_{\omega }^{N}$ can be expressed as.

4 $${A}_{\omega }^{N}=\sum_{m=0}^{M} \sum_{n=0}^{N} {D}_{N}{e}^{-i2\pi {\omega }_{N}({x}_{m}+{y}_{n})}{e}^{\pm 2\pi {\omega }_{N}{h}_{N}}$$

Let *h*_*N*_ = 0, Eq. (4) can be simplified as

5 $${\left({A}_{\omega }^{N}\right)}_{0}=\sum_{m=0}^{M} \sum_{n=0}^{N} {D}_{N}{e}^{-i2\pi {\omega }_{N}({x}_{m}+{y}_{n})}$$

Importing (5) into (4) yields

6 $${A}_{\omega }^{N}={\left({A}_{\omega }^{N}\right)}_{0}{e}^{\pm 2\pi {\omega }_{N}{h}_{N}}$$

The power spectrum ${E}_{\omega }^{N}$ is the square of the amplitude ${A}_{\omega }^{N}$, then the power spectrum ${E}_{\omega }^{N}$ can be expressed as

7 $${E}_{\omega }^{N}={\left({E}_{\omega }^{N}\right)}_{0}{e}^{\pm 4\pi {\omega }_{N}{h}_{N}}$$

Taking natural logarithms on both sides simultaneously, Eq. (7) simplifies to

8 $$\mathrm{ln}{E}_{\omega }^{N}=\mathrm{ln}{\left({E}_{\omega }^{N}\right)}_{0}\pm 4\pi {\omega }_{N}{h}_{N}$$

From the above equation, it can be seen that $\mathrm{ln}{E}_{\omega }^{N}$ is linearly related to ${\omega }_{N}$, so the field source burial depth h_*N*_ can be determined according to the bit-field spectrum theory^44–46^,The formula is as follows

9 $${h}_{N}=\frac{1}{4\pi }\frac{\Delta \mathit{ln}{E}_{\omega }^{N}}{\Delta {\omega }_{N}}(N=\mathrm{1,2},...,N)$$

In Eq. $\Delta \mathrm{ln}{E}_{\omega }^{N}$ and Δ ${\omega }_{N}$ are the rates of change of $\mathrm{ln}{E}_{\omega }^{N}$ and ${\omega }_{N}$.

In this paper, the study area is decomposed by DWT2D, and a total of five orders are decomposed. The corresponding depths of each order of wavelet details are 7.7 km, 11.1 km, 15.4 km, 23.9 km, and 42.0 km, respectively. The secondary decomposition of wavelet details of the 2nd-5th orders is carried out to obtain the finer 3D subsurface BGA. The depths of each order of wavelet details corresponding to the field sources are shown in Table 2.

**Table 2:** Corresponding field source depths for wavelet detail decomposition scales in the study area.

Gravity anomaly decomposition scale	Depth of field source (km)	Range of Bouguer gravity anomalies(mGal)
*D*_*1*_	7.7	-45.0 to 80.0
*D*_*2*_*D*_*1*_	10.1	-26.0 to 30.1
*D*_*2*_*D*_*2*_	11.8	-40.0 to 55.0
*D*_*2*_*D*_*3*_	13.1	-18.0 to 28.4
*D*_*2*_*D*_*4*_	13.5	-3.5 to 8.0
*D*_*3*_*D*_*1*_	14.5	-6.5 to 7.6
*D*_*3*_*D*_*2*_	17.4	-16.0 to 22.1
*D*_*3*_*D*_*3*_	19.1	-18.0 to 26.0
*D*_*3*_*D*_*4*_	19.5	-8.0 to 15.0
*D*_*4*_*D*_*1*_	20.0	-1.0 to 1.6
*D*_*4*_*D*_*2*_	24.8	-3.5 to 6.1
*D*_*4*_*D*_*3*_	27.0	-8.0 to 11.8
*D*_*4*_*D*_*4*_	36.7	-8.0 to 15.2
*D*_*5*_*D*_*1*_	38.9	-0.1 to 0.4
*D*_*5*_*D*_*2*_	45.4	-0.7 to 1.5
*D*_*5*_*D*_*3*_	49.8	-2.2 to 3.5
*D*_*5*_*D*_*4*_	52.9	-5.5 to 7.6

## Results and discussions

The findings of this study reveal the structural characteristics of the crust at varying depths within the research region.

### Distribution characteristics of the upper crustal structures

**Fig. 3:**
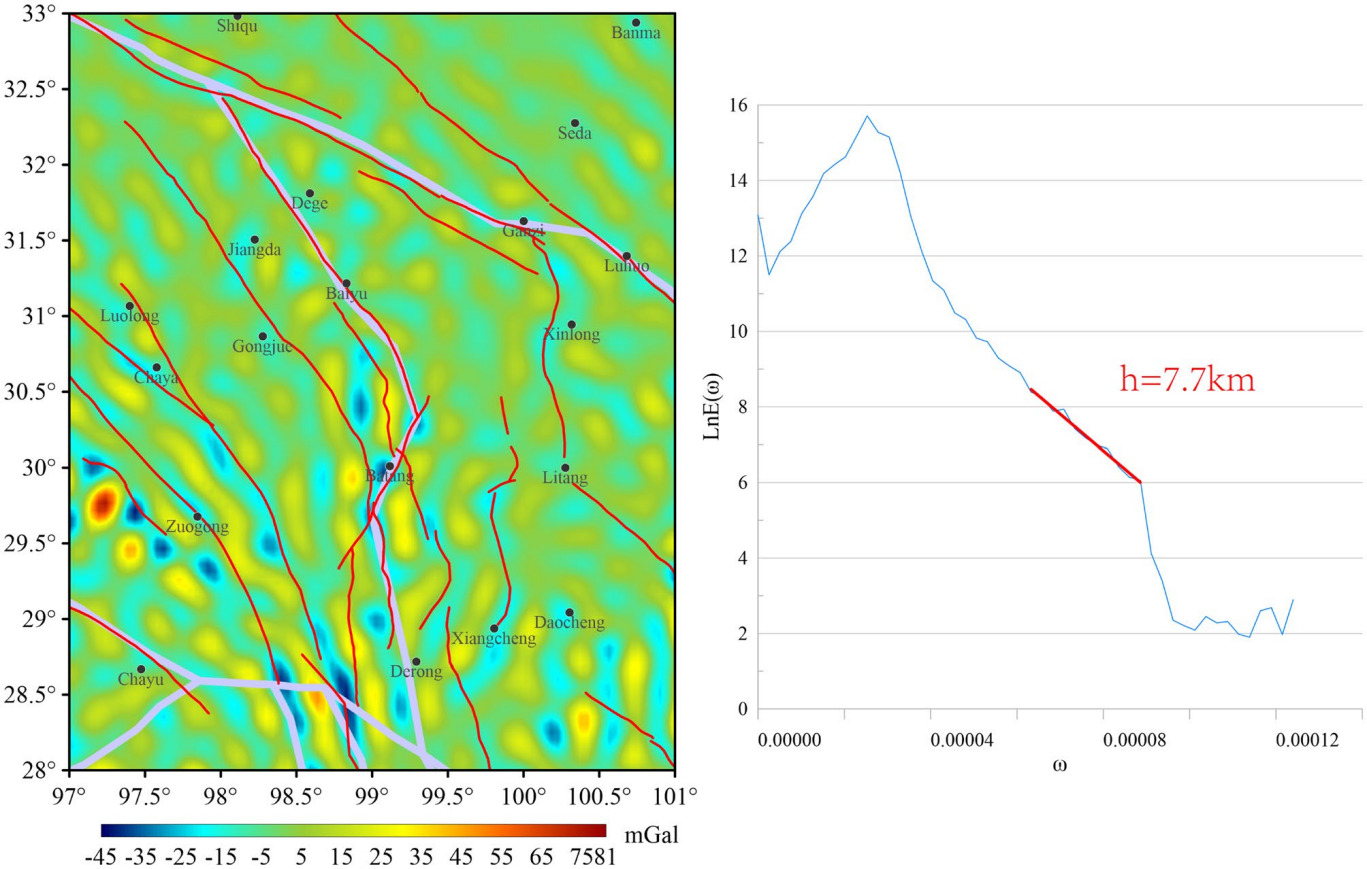
1st-order wavelet details and corresponding logarithmic power spectrum curves for the study area. The right plot has the frequency in the horizontal coordinate, the log power spectrum in the vertical coordinate, and the solid red line is the slope of the fit.

The range of BGA values of the 1st-order wavelet details is from -45 to 81 mGal (Fig. 3). The BGA transition zone remains indistinct, and the distribution of BGAs exhibits a scattered pattern, with a discernible interlacing of BGAs along the western side of F1, northwestward BGA extending along F3 and F4 to the east of Zuogong. Additionally, there are discernible low anomalies along the southern periphery of the Batang rupture and north–south striped low gravity anomalies on the left side of the southern part of F5. The relationship between the other fracture distribution and the gravity transition zone remains ambiguous. The BGA observed at these depths are found to be significantly influenced by surface topography and shallow sedimentary materials.

The distribution of BGA in the 1st-order wavelet decomposition is fragmented, and the trend of BGA transition zone is not obvious.

**Fig. 4:**
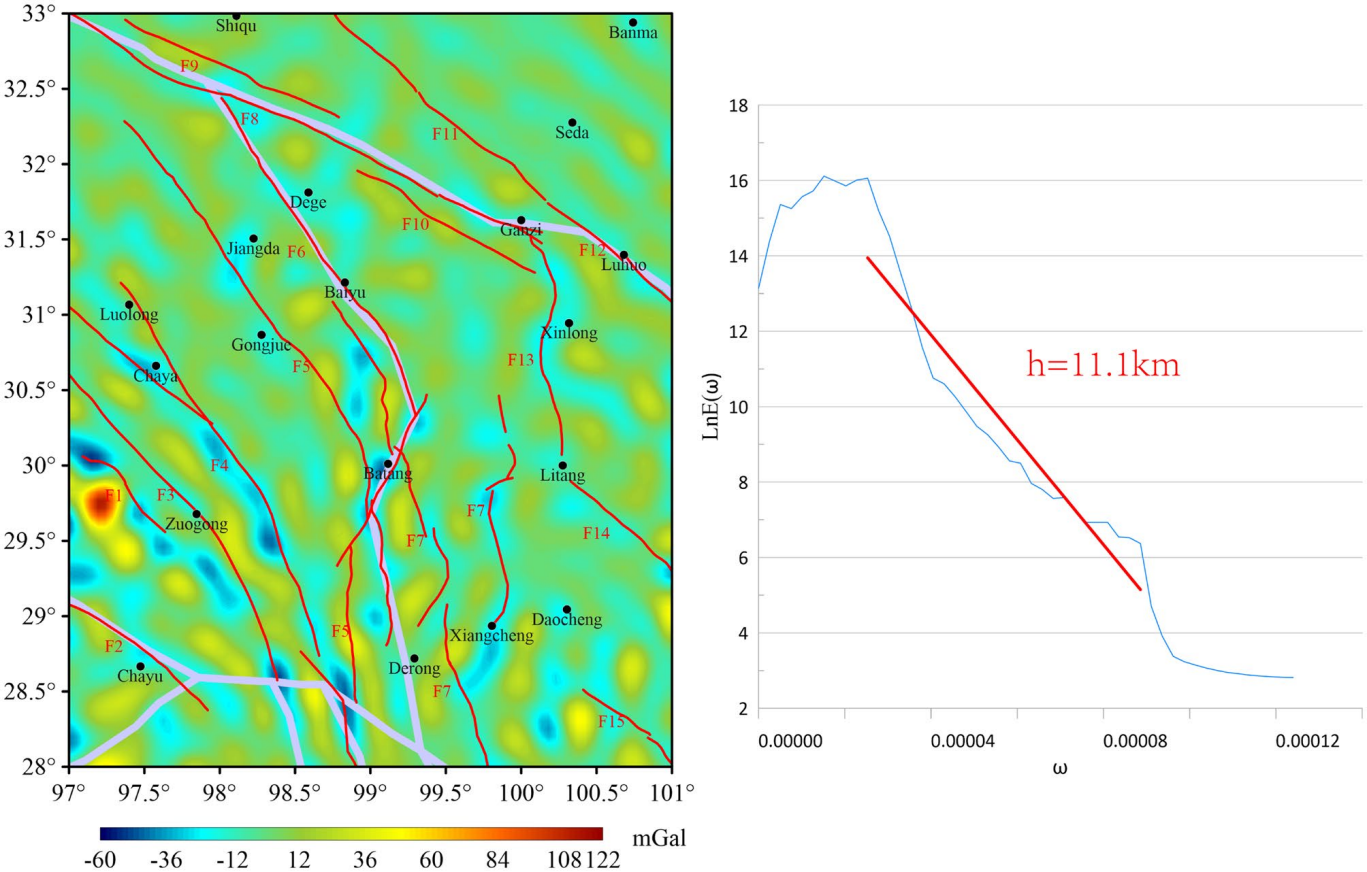
2nd-order wavelet details and corresponding logarithmic power spectrum curves for the study area.

The 2nd-order wavelet detail BGA amplitude in the study area ranges from -60 to 122 mGal. In comparison with the 1st-order, the BGA transition zone characteristics are more pronounced. The west side of Zuogong is identified as the area with high anomaly values (Fig. 4). A thorough examination of the 2nd-order wavelet details reveals that BGA transition zones are only discernible in the lower left quadrant. Each transition zone corresponds to F3, F4, F5, and along the Batang rupture from left to right, respectively. The estimated initial rupture depths of F3, F4, F5, and F6 range from 7.7 km to 11.1 km. It can be observed from the earthquake catalog in Table 3 that the focal depths of the two earthquakes that occurred along F6 on May 3, 1989 and August 12, 2013 were both 7 km. This focal depth aligns with the initial rupture depth of F6 obtained in this study, thereby validating the reliability of the present research. The BGA transition zone exhibits a correspondence with the three northwest-trending fractures (F8, F9, and F10) that are trending northwest on the northeast side of the study area.

**Table 3:** The seismic catalog of magnitude 4 and above in the study area. Data source: the China Earthquake Networks Center (http://10.5.160.18/console/index.action).

Time	Longitude(°E)	Latitude(°N)	Mag(Mw)	Depth(km)	Place
1722/-/-	99.10	30.00	6.00	-	Batang area, Sichuan
1747/03/-	100.70	31.40	6.75	-	Luhuo, Sichuan
1811/09/27	100.30	31.70	6.75	-	Zhuwo, Luhuo County, Sichuan
1878/12/-	97.50	28.50	6.50	-	Chayu, Tibet
1911/07/-	97.50	28.50	6.50	-	Chayu, Tibet
1919/05/29	100.50	31.50	6.25	-	Northwest of Daofu, Sichuan
1919/08/26	100.00	32.00	6.25	-	Ganzi area, Sichuan
1920/12/22	98.50	29.00	6.00	-	Mangkang, Tibet
1923/10/20	99.00	30.00	6.50	-	Near Batang, Sichuan
1930/04/28	100.00	32.00	6.00	-	North of Ganzi, Sichuan
1951/03/17	97.40	30.90	6.00	-	Near Qamdo, Tibet
1967/08/30	100.30	31.60	6.80	-	Northwest of Luhuo, Sichuan
1973/02/08	100.33	31.70	6.00	-	Northwest of Luhuo, Sichuan
1979/03/29	100.50	31.60	6.00	-	Northwest of Luhuo, Sichuan
1982/06/16	97.30	32.40	6.20	45	Southeast of Yushu, Qinghai
1989/04/16	100.03	31.96	6.00	15	Northwest of Ganzi, Sichuan
1989/04/25	99.23	29.99	6.60	12	Southeast of Batang, Sichuan
1989/05/03	99.42	30.05	6.60	7	East of Batang, Sichuan
1989/05/03	99.54	30.11	6.30	14	East of Batang, Sichuan
2013/08/12	99.55	30.07	6.30	7	East of Batang, Sichuan
1816/12/08	97.96	30.05	6.10	10	Zuogong County, Qamdo, Tibet
1870/04/11	100.70	31.40	7.50	-	Luhuo, Sichuan
1896/03/-	99.10	30.00	7.25	-	Batang, Sichuan
1923/03/24	98.00	32.50	7.00	-	Luoxu, Shiqu County, Sichuan
1948/05/25	101.00	31.50	7.30	12	Between Luhuo and Daofu, Sichuan
1973/02/06	100.50	29.50	7.60	17	Litang, Sichuan

**Fig. 5:**
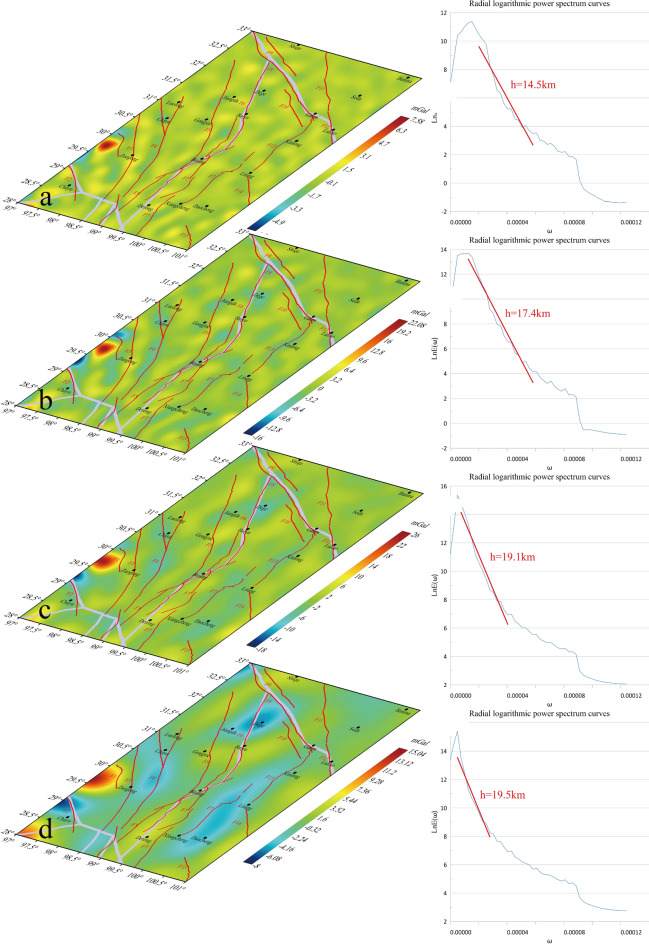
Corresponding depths of secondary decomposition details for 2nd-order details.

The overall effect of the 2nd-order wavelet quadratic decomposition details should be equivalent to the 2nd-order wavelet details. That is to say, *D*_*2*_ = *D*_*2*_*D*_*1*_ + *D*_*2*_*D*_*2*_ + *D*_*2*_*D*_*3*_ + *D*_*2*_*D*_*4*_. The corresponding depths of the field sources for each order wavelet detail are 10.1 km, 11.8 km, 13.1 km, and 13.5 km, respectively (Fig. 5).

In the* D*_*2*_*D*_*1*_ (Fig. 5a) detail map, F1, F3, and F4 are distributed along the BGA low value zones. Therefore, it is hypothesized that the rupture initiation depths of F1, F3, and F4 are around 10.1 km. The F3 is in the top region of the eastern Himalayan tectonic nodal arc, which is affected by the remote effect of lateral extrusion of the QTB and collisional extrusion of the Indian Plate and the Eurasian Plate. The magnitude 6.1 earthquake that occurred north of Zuogong on December 8, 1816, had a focal depth of 10 km. This earthquake is located on F4, which is basically consistent with the initial rupture depth of 10.1 km of the F4 fault obtained earlier. The overall trend of the *D*_*2*_*D*_*2*_(Fig. 5b) wavelet details is not significant compared to the *D*_*2*_*D*_*1*_(Fig. 5a) details. However, the distribution of negative anomalies in the study area is more pronounced in the *D*_*2*_*D*_*2*_(Fig. 5b) order than in the *D*_*2*_*D*_*1*_(Fig. 5a) order. Therefore, the BGA transition zone are also more pronounced. The detailed map of *D*_*2*_*D*_*2*_(Fig. 5b) wavelet decomposition reveals a BGA transition zone with high and low values in the southern part of the study area, situated between Chayu and Derong. This transition zone aligns with the distribution of F5 and F7 in this region. The F7 is part of the JZRSZ, which is situated between the QTB and the Northwest Sichuan Block. This fracture zone comprises six to seven primary fractures, with a total length of approximately 700 km and a width of about 80 kilometers^13^. The F6, F7, F10, F12, F13, F14, and F15 initially correspond to the BGA transition zone at this discretization scale. This zone corresponds to a field source buried at a depth of 11.8 km. The F10, a fracture within the landmass, is distributed along the southern part of F8. However, its activity is much lower than that of F8. The magnitude 6.6 earthquake on April 25, 1989 occurred on F7 east of Batang, with a focal depth of 12 km, which is consistent with the rupture depth of F7 obtained earlier. The trend information provided by the *D*_*2*_*D*_*3*_(Fig. 5c) order wavelet detail information is particularly evident, exhibiting two distinct BGA transition zone along the left boundary of the study area, corresponding to the F1 and F2 distributions, respectively. The BGA on the west side of F1 exhibits a high value of 28.4 mGal, which is the most significant value observed in the study area at this wavelet decomposition scale. The southwest side of the site marks the lower boundary of the QTB, where a portion of the crust exists in a molten state while exhibiting signs of hardening. Consequently, the BGA manifests a transition zone that exhibits both high and low values^19^. Batang is situated within a region of low anomaly, occupying a space in the northern SYB. The Batang Fault, located within Batang, is characterized as a dextral strike-slip fault. The presence of low value BGA in this area may be attributed to the dextral strike-slip, which has resulted in the removal of a portion of the underlying material. Furthermore, F3, F4, F6, F7, F9, F10, F13, F14, and F15 exhibit anomalous distribution along the transition corresponding to the *D*_*2*_*D*_*3*_(Fig. 5c) detail. This observation suggests the hypothesis that the aforementioned fractures persistently rupture at the *D*_*2*_*D*_*3*_(Fig. 5c) decomposition scale, corresponding to the burial depth of the source of the field of 13.1 km. The depth of *D*_*2*_*D*_*4*_(Fig. 5d) corresponds to 13.5 km, as determined by the radial log power spectrum results. The BGA exhibits discernible local features. The BGA on the right side of Batang exhibits a broad spectrum of low values, thereby substantiating the hypothesis that the right-hand rotation of the Batang rupture has dislodged a portion of the subterranean material.

**Fig. 6:**
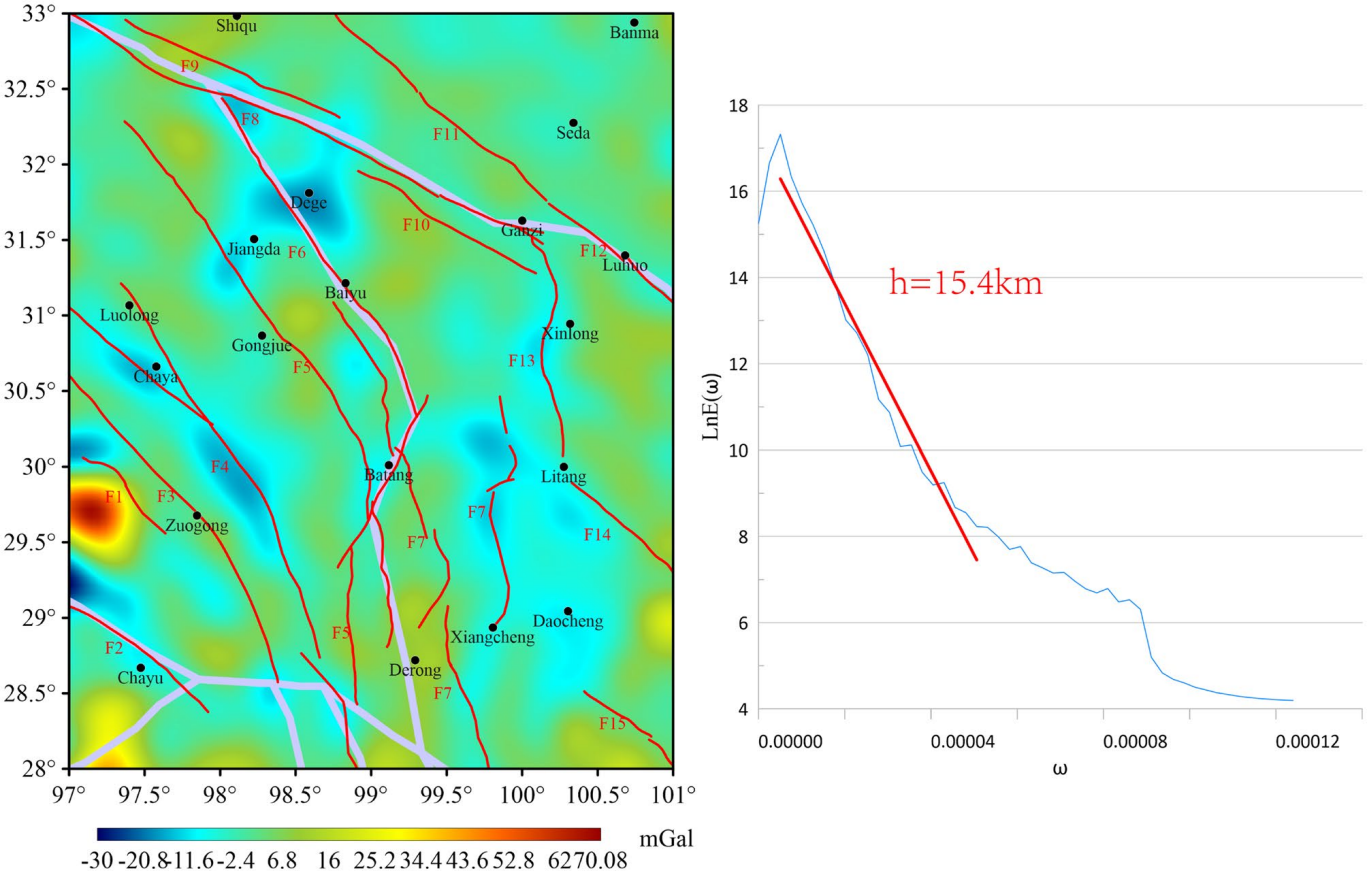
3rd-order wavelet details and corresponding logarithmic power spectrum curves for the study area.

**Fig. 7:**
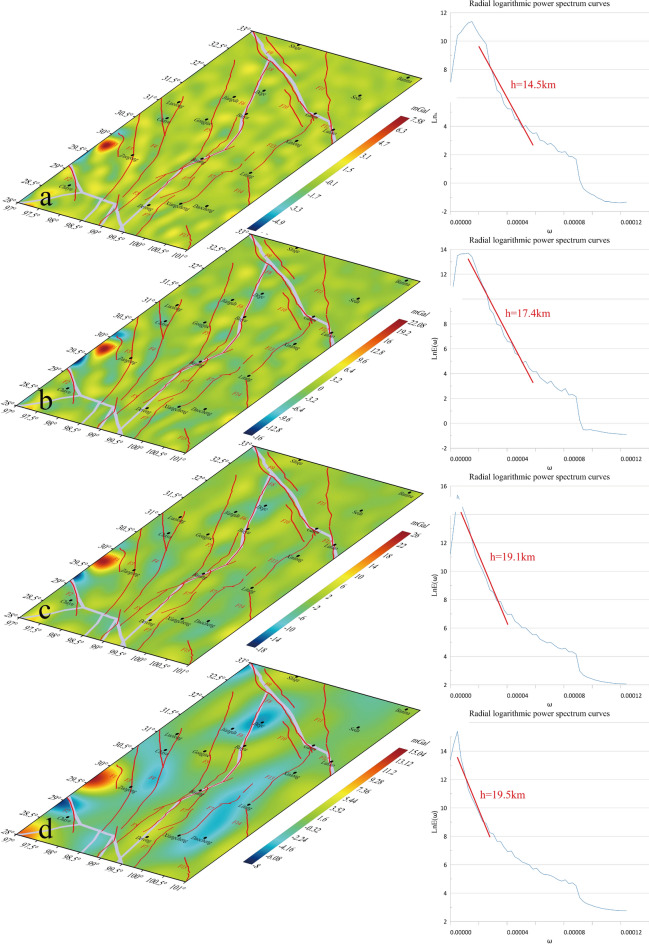
Corresponding depths of secondary decomposition details for 3rd-order details.

The 3rd-order wavelet details of the study area (Fig. 6) demonstrate that the BGA amplitude ranges from -30 mGal to 70 mGal, with the highest value also located in the western part of Zuogong. The high value of BGA exhibits a complete closed curve. This range exceeds that of the 2nd-order wavelet details (Fig. 6). The positive values of the BGA are indicative of the distribution of high-density material. This suggests that the deeper and denser mantle material at this location spreads eastward with increasing depth^19^. The distribution of both F1 and F2 is coincident with the BGA high-low transition zone. The northern portion of F2 exhibits the lowest value of -30 mGal within the region. The upper section of F6 is distributed along the BGA low value near Dege, and the middle and lower sections are distributed along the BGA transition zone near Batang. The F13 and F14 are distributed along the BGA transition zone at 3rd-order detail. The burial depths corresponding to the secondary decomposition of the 3rd-order wavelet detail are 14.5 km, 17.4 km, 19.1 km, and 19.5 km, respectively (Fig. 7).

The BGA transition zone is not readily apparent in the *D*_*3*_*D*_*1*_(Fig. 7a) order wavelet detail map. In *D*_*3*_*D*_*2*_(Fig. 7b), with the exception of F8, the remaining ruptures exhibit a stronger correspondence to the BGA transition zone. *D*_*3*_*D*_*3*_(Fig. 7c) demonstrates a comparable distribution of results, suggesting that the rupture depths exceed 19.1 km. It can be seen from Fig. 6 that there have been three earthquakes of magnitude 6 or above in the east of Batang in history, all located in the gravity anomaly transition zone. Among them, the focal depth of the magnitude 6.3 earthquake on May 3, 1989 was 14 km. This depth is consistent with the range of the secondary decomposition of the third-order wavelet decomposition, and the corresponding F7 also exists at this depth.

At the *D*_*3*_*D*_*4*_(Fig. 7d) scale, the northern section of F6 obliquely cuts the region of low value anomalies near Dege, suggesting the existence of a low-density body in this area and that the rupture depth of the northern section of the rupture has terminated at this scale, corresponding to a depth of 19.5 km. Furthermore, a high value anomaly of considerable value has been identified on the southern periphery of Chayu at the *D*_*3*_*D*_*4*_(Fig. 7d) scale. This finding indicates the potential for multiple channels responsible for the eastward migration of deep material. This observation is in alignment with the conclusions drawn by Zhuo et al^47^.

### Distribution characteristics of the middle crustal structures

**Fig. 8:**
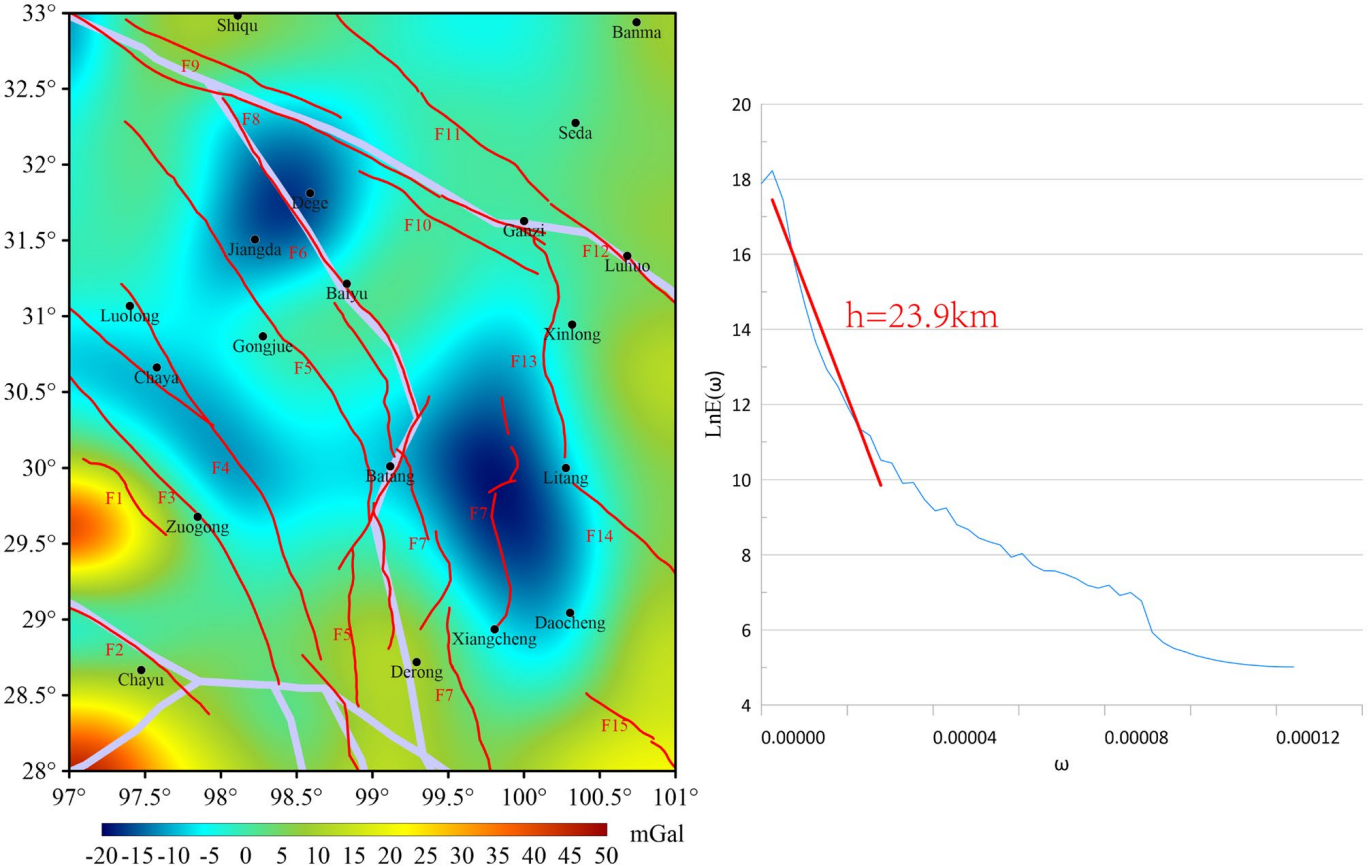
4th-order wavelet details and corresponding logarithmic power spectrum curves for the study area.

**Fig. 9:**
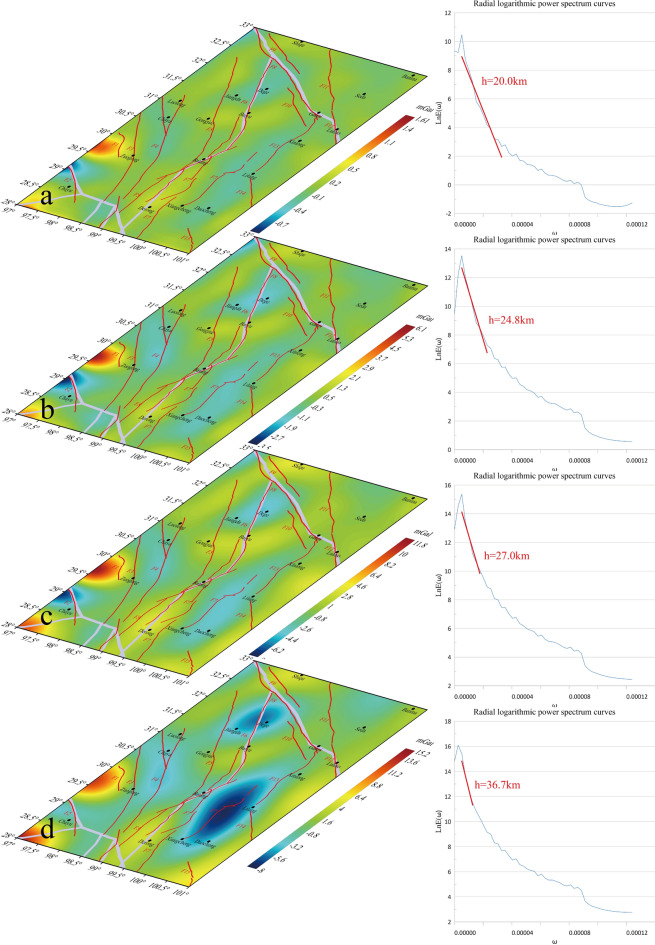
Corresponding depths of secondary decomposition details of 4th-order details.

As illustrated in the 4th-order wavelet detail map of the study area (Fig. 8), the overall low value anomalies are predominantly concentrated within the range of -20 to 50 mGal. The distribution of high-density bodies on the western side of Zuogong exhibits an expansion, and the high-value anomalies in the southern part of Chayu demonstrate an extension in an eastward direction. This indicates that the deeper material flows in an eastward direction. The overall distribution of BGA exhibits a trend of high values in the southwest and low values in the northeast. Additionally, two regions of low value anomaly are identified on a global scale, with a diagonal distribution in the northwest and southeast. The radial log power spectrum depth of the 4th-order wavelet is 23.9 km, and the depths of the quadratic decompositions are 20.0 km, 24.8 km, 27.0 km, and 36.7 km, respectively (Fig. 9). The 4th-order detail reveals a substantial array of low value anomalies in the northern SYB suggesting the presence of low-density material or partial melting of the crust in this region. This finding is consistent with the results of a study conducted by Hui et al. in the same area. The study’s 20-km velocity level slices of the S-wave subsurface, as analyzed by Sun et al., demonstrate the existence of a low-velocity zone of the S-wave in this area, indicative of crustal partial melting in this region^48^.Given that the decomposition depth of the *D*_*4*_*D*_*4*_(Fig. 9d) wavelet details approaches 40 km, this depth falls within the mid-crustal range defined by CRUST1.0 and aligns closely with CRUST1.0 findings, thereby revealing significant deep crustal information. This finding suggests that these anomalies experience minimal influence from shallow sediments at this decomposition scale and are predominantly driven by mid-crustal upheaval.

The overall effect of the 4th-order wavelet decomposition details is consistent with the effect of the secondary decomposition, i.e., *D*_*4*_ = *D*_*4*_*D*_*1*_ + *D*_*4*_*D*_*2*_ + *D*_*4*_*D*_*3*_ + *D*_*4*_*D*_*4*_.From* D*_*4*_*D*_*1*_(Fig. 9a), the overall BGA of the study area are near the zero value, and except for the two fractures in the northern sections of F4 and F6, the rest of the fractures are still located in the BGA transition zone, and this result is more obvious in* D*_*4*_*D*_*2*_(Fig. 9b). The fracture detected in the non-converted zone of *D*_*4*_*D*_*2*_(Fig. 9b) corresponds to the small rightmost fracture of F7, suggesting that this fracture is no longer observed at that depth. The *D*_*4*_*D*_*2*_(Fig. 9b) corresponds to a depth of 24.8 km, and the *D*_*4*_*D*_*3*_(Fig. 9c) situation is overall consistent with *D*_*4*_*D*_*2*_(Fig. 9b).

In the* D*_*4*_*D*_*4*_(Fig. 9d) detail map, F3, F5, F6 middle, F7 left small fracture, F8, F11, and F12 coincide with the BGA transition zone boundary, indicating that all of these fractures are present at this scale. Furthermore, *D*_*4*_*D*_*4*_(Fig. 9d) corresponds to a depth of 36.7 km.

### Distribution characteristics of the lower crustal structures

**Fig. 10:**
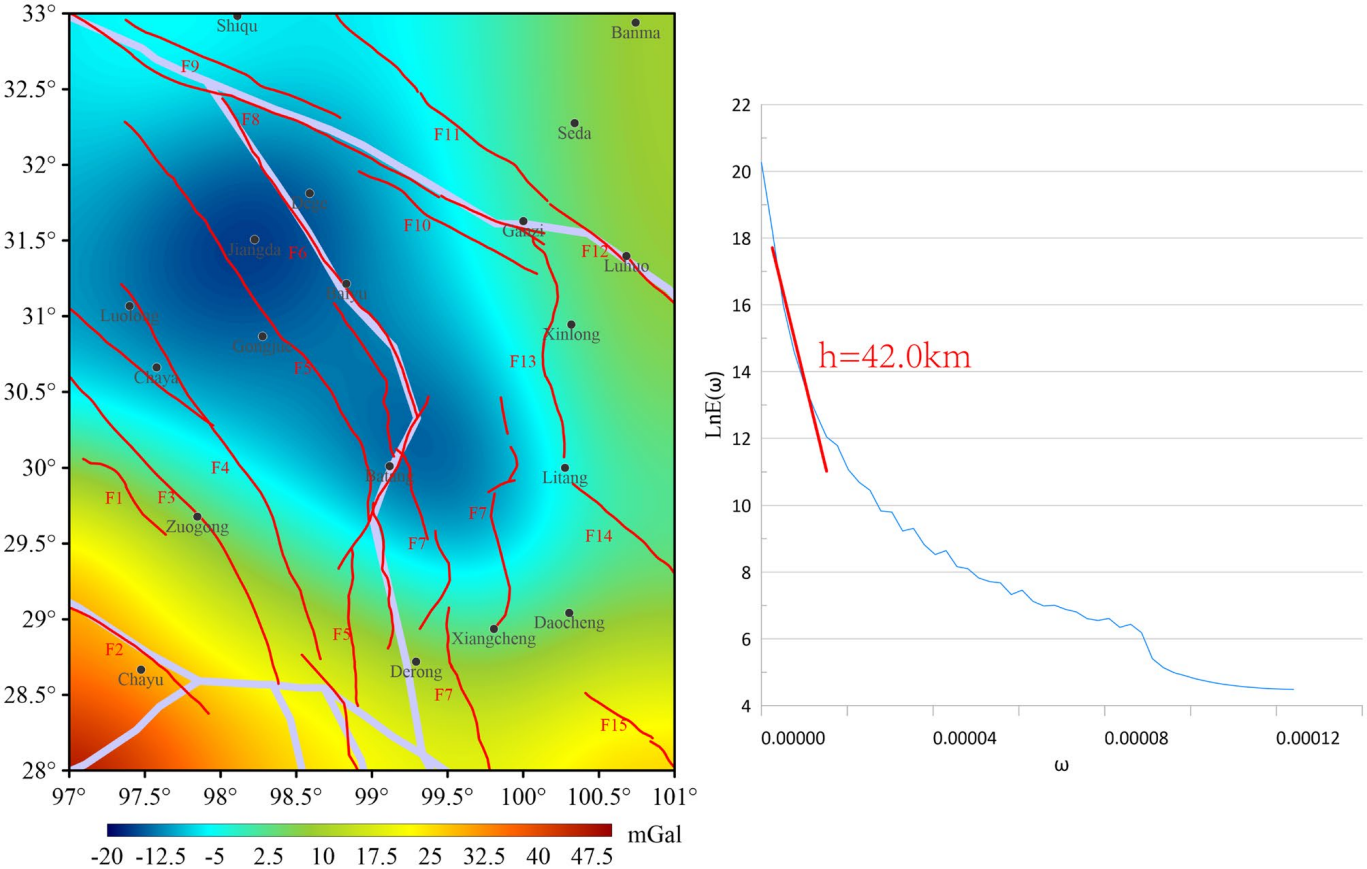
5th-order wavelet details and corresponding logarithmic power spectrum curves for the study area.

In this study, the CRUST1.0 model is employed to calculate the depth of the lower crust in the study area to be 47–68 km. The pattern of change in these crustal depths is found to be analogous to the pattern of the 5th-order wavelet detail distribution features in this study. The radial logarithmic power spectrum of the 5th-order details in the study area corresponds to a field source burial depth of 42 km, which aligns with the findings of previous studies and CRUST1.0 model data. As illustrated in the 5th-order wavelet detail decomposition map (Fig. 10), the region is predominantly characterized by low value anomalies, exhibiting an overall amplitude range of -20 to 50 mGal. The F3 location persists within the BGA transition zone, suggesting the potential of F3 as a deep major fracture. The F3 has experienced heightened levels of stress accumulation, suggesting an elevated probability of future seismic events with magnitudes exceeding 6.5 on the Richter scale. At the 5th-order wavelet detail scale, F7, F12, and F13 are also situated within the BGA transition zone, indicating a high likelihood of these ruptures occurring at depth and magnitude. The sedimentary layers, upper middle, and lower crustal depth ranges in the region in the CRUST1.0 model data are 0.4 ~ 4.4 km, 20.5 ~ 31.5 km, 35 ~ 49 km, and 47 ~ 68 km, respectively. A comparison of the wavelet detail field source burial depths in this paper with the upper middle and lower crustal depth ranges in the CRUST1.0 model demonstrates that this paper can provide more detailed structural on the distribution of the crust.

**Fig. 11:**
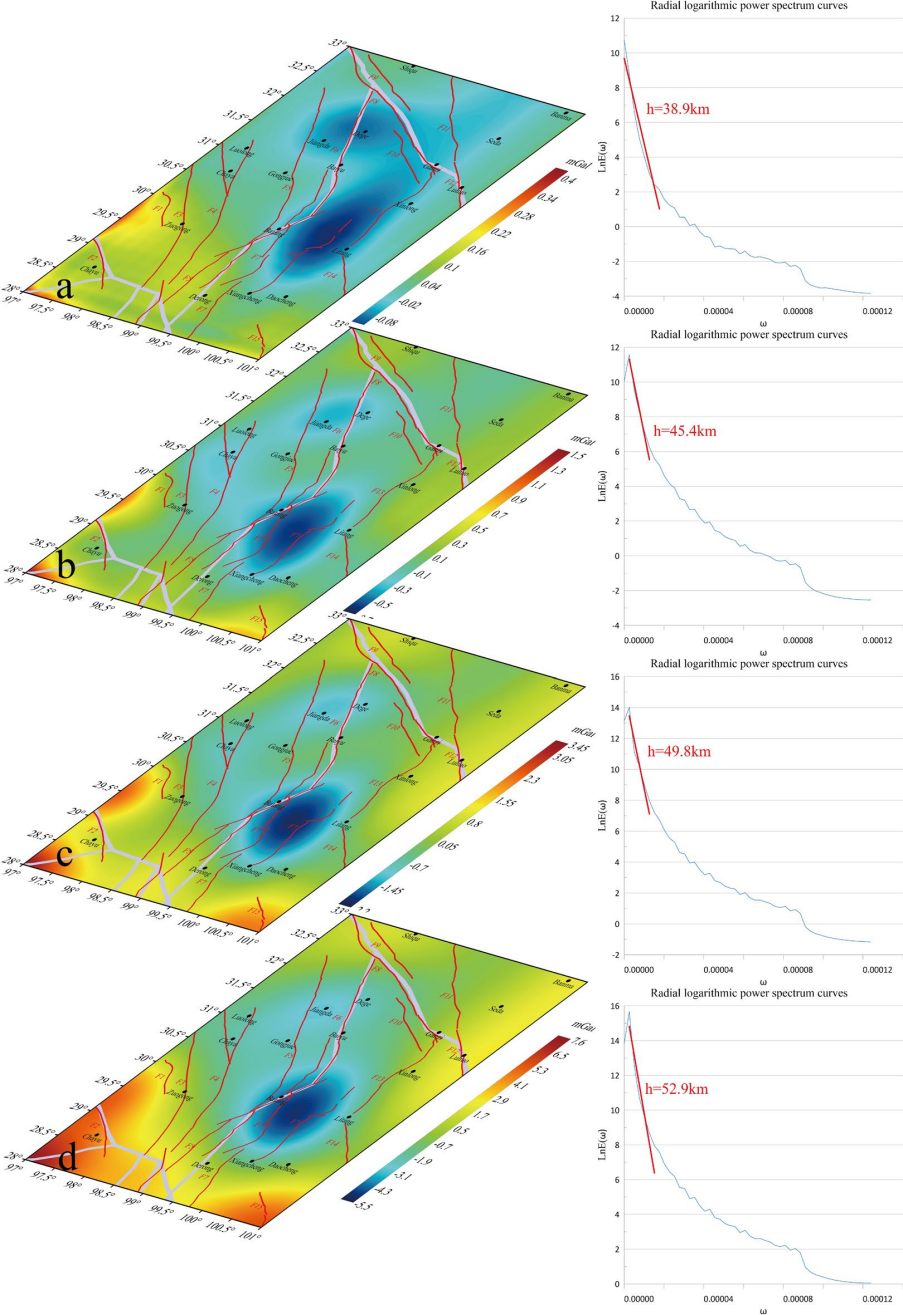
Corresponding depths of secondary decomposition details of 5th-order details.

The anomalies corresponding to *D*_*5*_*D*_*1*_, *D*_*5*_*D*_*2*_, *D*_*5*_*D*_*3*_, and *D*_*5*_*D*_*4*_ in Fig. 11 are located at depths of 38.9 km, 45.4 km, 49.8 km, and 52.9 km, respectively. The CRUST1.0 model indicates a depth range of 35–68 km for the middle and lower crust. This suggests that the BGA observed at this scale are primarily attributable to upheavals within the middle and lower crustal regions. Consequently, detailed maps of *D*_*5*_*D*_*2*_-*D*_*5*_*D*_*4*_ exhibit no discernible changes. Consequently, the initial depth of the lower crust is estimated to range from 36.7 km to 38.9 km. According to Zhang’s findings, the range of the lower crust is between 58 and 62 km, respectively. This paper posits that the initial depth of the lower crust should be calculated from 36.7 to 38.9 km, with the intermediate value of 37.8 km being selected to ensure the final depth of the lower crust is between 37.8 and 68 km^49^.The magnitude 6.2 earthquake that occurred in Yushu, Qinghai Province on June 16, 1982, had a focal depth of 45 km underground. It can be observed from Fig. 11b that this earthquake is located in the BGA transition zone. The corresponding depth of *D*_*5*_*D*_*2*_ is 45.5 km, and the epicenter is near F5.

The study area contains 15 identified fractures exhibiting a depth-dependent distribution from shallow to deep structural layers. This investigation specifically examines seven major faults (F1, F3, F4, F6, F7, F8, F13), with their corresponding termination field sources demonstrating average burial depths of 38.9 km (F1/F8), 52.9 km (F3/F4/F6/F7/F13). Notably, the depth measurements reveal two distinct burial depth clusters at approximately 38.9 km and 52.9 km.

These seven faults are all active faults without hidden faults, all of which have been active since the Quaternary and may continue to be active in the future. Furthermore, all of these faults are deep and large, and their depths are within the lower crust of the CRUST1.0 model.

## Conclusions

This paper utilizes a gravimetric approach to conduct an in-depth exploration of the southeast margin of the TP, a region of significant geological interest. The rupture depths of the primary ruptures in the study area are precisely analyzed by using DWT2D, in conjunction with field source separation theory, through the analysis of the BGA field data. This study makes two primary contributions. First, it provides a comprehensive understanding of the complex geological and tectonic features of the region. Second, it offers significant data supporting the geological evolution of the southeast margin of the TP, which is of both scientific significance and practical importance.

- (1) Two wavelet decompositions of the BGA can not only more accurately determine the rupture depth of the fault, but also significantly improve the identification of shallow structures. Effective decoupling of deep and shallow superposition anomalies. The logarithmic power spectrum analysis using log power spectral methods can improve the resolution of weak anomalous signals at depth. This is particularly important for the identification of deeply buried basement formations.
- (2) This study elucidates the subsurface depth-dependent structural contacts of faults through precise analysis of power spectra derived from DWT2D.Comparison with the CRUST1.0 model reveals that the depth range of the lower crust is 37.8–68 km. This finding provides two significant benefits. First, it serves as an important reference for understanding the crustal structure of the region. Second, it lays a scientific foundation for future geological research and resource exploration in the area. It can be seen that historical earthquakes of magnitude 6 or above in the study area are almost all distributed in the fault and its adjacent areas. Therefore, studying the elongation depth of faults is of great significance for the research related to the causes of earthquakes.
- (3) The depth distribution of the fractures in this study area ranges from shallow to deep. Among them, the seven major fractures are classified as deep fractures, with depths located within the lower crust, as defined by the CRUST1.0 model. According to the seismic network’s source depth data, it is evident that the source depths of the recorded earthquakes occurring on faults fall entirely within the rupture depth range obtained in this study. In terms of the strike, most fractures, predominantly exhibit a northwesterly orientation, with representative examples such as F3, F4, F8, etc. However, fractures F6 and F7 deviate from this general orientation.

## Data Availability

The datasets generated and/or analysed during the current study are available in the Zenodo repository, 10.5281/zenodo.15164995
